# Mechanical, Thermal, and Microstructural Characterization of FDM-Printed PLA/Obsidian Composites

**DOI:** 10.3390/polym18131563

**Published:** 2026-06-23

**Authors:** Fatih Alibeyoglu

**Affiliations:** Department of Mechanical Engineering, Kafkas University, Kars 36100, Türkiye; falibeyoglu@kafkas.edu.tr

**Keywords:** polylactic acid (PLA), obsidian, volcanic glass filler, FDM/FFF, 3D printing, mechanical properties, thermal stability, fractography, SEM, interfacial coupling

## Abstract

FDM-printed polylactic acid (PLA) composites containing 5 and 10 wt% obsidian powder sourced from the Kars region of Eastern Anatolia (Turkey) were produced via twin-screw masterbatch extrusion and subsequent single-screw filament dilution. Mechanical (tensile, three-point flexure, notched Charpy impact, Shore D), physical (density), thermal (simultaneous TGA/DSC) and microstructural (macroscopic fractography and SEM at 100×–1000×) characterizations were performed on FDM-printed specimens. Young’s modulus rose monotonically by +9.0% at 5 wt% and +18.2% at 10 wt%, while ultimate tensile strength decreased by 12.4% and 17.3%, respectively. The flexural modulus increased by +15.2% at 5 wt% and plateaued at 10 wt% (+16.7%), whereas the flexural strength decreased by only 3.5% at 10 wt%, indicating that flexure-mode loading is markedly more tolerant of obsidian filler than axial tension. Shore D hardness rose by +2.11 points from 0 to 5 wt% with saturation thereafter. TGA showed a dual thermal effect: T_5_ and T_10_ dropped by 5–6 °C from 5 to 10 wt%, while the main decomposition rate decreased by ~46% and the decomposition interval widened from 9.7 to 23.5 °C, indicating a barrier/heat-shielding effect of dispersed silicate particles. SEM revealed a continuous ductile → transitional → brittle progression with increasing obsidian content; extended interfacial debonding lines at 10 wt% identified weak unmodified filler/matrix coupling as the principal performance-ceiling factor. Density measurements indicated a ~3–6% residual void fraction consistent with the inter-bead voids observed by SEM. To the authors’ knowledge, this is the first systematic study of obsidian as a reinforcing filler in PLA; the 5 wt% composition is identified as a strong candidate for esthetic, flexure-dominant, and low-load structural applications.

## 1. Introduction

Polylactic acid (PLA) is a bio-based and biodegradable thermoplastic polymer obtained from renewable sources (corn starch, sugarcane, etc.). Owing to its moderate mechanical strength, good processability, and low environmental impact, PLA has become one of the most widely used materials in additive manufacturing technologies such as Fused Deposition Modelling (FDM)—also known as Fused Filament Fabrication (FFF) [1]. However, the brittleness, limited thermal resistance, and modest stiffness of neat PLA constrain its use in engineering applications requiring high mechanical performance. To overcome these limitations, the development of composite materials by incorporating various mineral and ceramic fillers into the PLA matrix has emerged as an active research area.

The principal motivations for adding mineral fillers to PLA include increasing the stiffness and elastic modulus, improving thermal stability, reducing production cost, and imparting functional properties [2]. Mineral fillers are typically low-cost, abundant, and environmentally benign materials, offering both economic and sustainability advantages [3,4]. In recent years, studies on mineral-reinforced PLA composites for FDM/FFF have proliferated, and the properties of composites obtained with various inorganic fillers have been examined in detail.

Glass fillers are among the most widely used mineral reinforcements in PLA composites. Vidakis et al. integrated glass fillers in powder, bead, and flake forms into a PLA matrix at three different loadings, reporting up to 60% reinforcement under flexural loading, particularly in the flake form [5]. Efstratiadis et al. likewise showed that in PLA composites filled with recycled glass beads, the elastic modulus rose by up to 16%, the flexural modulus by up to 23%, and the flexural strength by up to 11%, while tensile strength and elongation decreased [1]. For ceramic fillers, Hedjazi et al. [2] showed that commercially available ceramic-reinforced PLA filaments could be successfully processed by FDM in horizontal and lateral print orientations, with only a 9% reduction in stiffness relative to the filament properties [2]. For hydroxyapatite (HAp) fillers—prominent in biomedical applications—Begum et al. reported an approximately 6% rise in Shore D hardness at 10 wt% loading [6]. Overall, the addition of mineral fillers to PLA matrices tends to increase the modulus and hardness, frequently decreases the tensile strength, and affects the thermal stability to varying extents [1,2,3,4,5,6]. Nevertheless, no studies have been encountered in the literature on obsidian-filled PLA composites.

Obsidian is a naturally amorphous volcanic glass formed by the rapid cooling of magma without time for crystallization. Chemically, it is silica-dominated (~70–75% SiO_2_), with significant alumina (~12% Al_2_O_3_), Na_2_O, K_2_O, and Fe oxides, and produces extraordinarily sharp edges through conchoidal fracture—historically used for cutting tools and modern scalpel blades [7]. Reported physical properties of obsidian include a density of 2.3–2.5 g/cm^3^, a Mohs hardness of 5–6, comparable to silica glass, chemical durability similar to borosilicate glass, and Young’s modulus values in the 60–80 GPa range from nano-indentation studies [7,8]. These properties make obsidian conceptually attractive as a polymer filler: it is hard, rigid, chemically inert, naturally amorphous (with no crystalline transitions in the polymer-processing range), and abundant in geologically active regions. The Kars region of Turkey is one of Anatolia’s important obsidian sources; the Kars plateau hosts multiple Late-Cenozoic rhyolitic obsidian outcrops that have been geochemically catalogued in archaeometric literature [9]. The hardness, glassy structure, and natural abundance of obsidian make it an interesting candidate filler material for PLA composites. However, no study exists in the literature on the use of obsidian as a reinforcement in a PLA matrix. To the authors’ knowledge, this study is the first systematic investigation of the use of obsidian as a reinforcing filler for PLA.

Beyond the choice of filler, the mechanical response of FFF-printed PLA is strongly governed by its thermal and structural history: PLA processed by fused filament fabrication undergoes time-, temperature- and structure-dependent physical ageing and crystallinity evolution that modify its subsequent mechanical behaviour [10]. Distinguishing the intrinsic effect of a filler from this background sensitivity therefore requires both a matched neat-PLA reference and explicit reporting of processing and printing conditions—a requirement addressed in the present study.

The aims of this study are: (1) to produce PLA composite filaments containing 5 and 10 wt% Kars obsidian powder via twin-screw extrusion; (2) to fabricate test specimens via FDM; (3) to characterize the mechanical properties (tensile, flexural, impact, hardness); (4) to characterize the physical properties (density); (5) to characterize the thermal properties (TGA, DSC); and (6) to analyze the fracture morphology (SEM). By revealing the properties of obsidian-reinforced PLA composites, this study aims to evaluate the engineering potential of this new material system.

## 2. Materials and Methods

### 2.1. Materials

A commercial polylactic acid (PLA) filament was used as the matrix material. The PLA filament was supplied with a diameter of 1.75 mm and was dried at 80 °C for 12 h prior to extrusion to reduce its moisture content.

**Figure 1:**
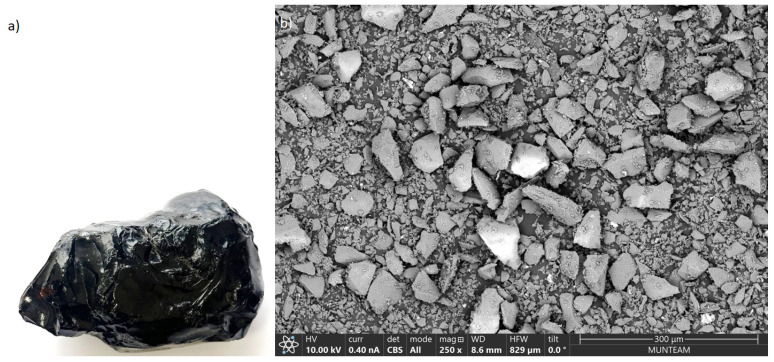
The Kars-region obsidian filler: (**a**) raw obsidian sample as collected; (**b**) SEM micrograph (backscattered-electron, 250×) of the ball-milled powder (<44 µm fraction), showing angular fragments bounded by conchoidal fracture surfaces over a broad size range. Scale bar in (**b**) =300 µm.

For preparing the obsidian powder, raw obsidian fragments were first subjected to mechanical milling using a ball mill to reduce the obsidian pieces to small sizes. After milling, the obsidian powder was sieved through 44 µm mesh openings to obtain the fraction with particle size < 44 µm (Figure 1). The sieving step ensured homogeneity of the particle size distribution and better dispersion within the composite. The obtained obsidian powder was dried at 105 °C for 24 h prior to extrusion to minimize moisture content.

The phase structure of the obsidian powder was characterized by X-ray diffraction (XRD) using a Rigaku MiniFlex diffractometer (Rigaku Corporation, Akishima, Tokyo, Japan) with Cu Kα radiation (λ = 1.5406 Å) operated at 40 kV and 15 mA. Patterns were collected over a 2θ range of 10–90° in continuous Theta/2-Theta mode at a scan speed of 2°/min and a step width of 0.02°, using a bent diffracted-beam monochromator and an SC-70 detector.

Functional group analysis was carried out by Fourier-transform infrared spectroscopy (FTIR) in the 4000–400 cm^−1^ region for neat PLA, the 5 and 10 wt% composites, and obsidian powder. Spectra were recorded using a Bruker FTIR spectrometer (Bruker Optik GmbH, Ettlingen, Germany) equipped with a single-reflection diamond ATR (Platinum ATR) accessory, at a spectral resolution of 4 cm^−1^.

The morphology, size, and shape of the milled obsidian powder were characterized by SEM (backscattered-electron mode, 10 kV) combined with image analysis morphometry. Individual particles were segmented with watershed deagglomeration of contacting fragments, and the equivalent-circle diameter, circularity, and aspect ratio of 1446 particles (retained on a minor-axis ≤ 44 µm criterion consistent with the sieve cut-off) were measured across replicate 1000× fields.

### 2.2. Composite Filament Production

#### 2.2.1. Preparation of the PLA/Obsidian Masterbatch

PLA and obsidian powder were compounded using a twin-screw extruder. The masterbatch composite contained 20 wt% obsidian powder and 80 wt% PLA. To ensure homogeneous dispersion and stable melt flow, a six-zone temperature profile was applied during extrusion. The temperature zones, from feed to die exit, were set to 165, 172, 180, 188, 195, and 200 °C. After extrusion, the extrudate was cooled, pelletized, and prepared for filament production. The recorded melt pressure during masterbatch compounding was approximately 12 MPa, with a corresponding specific screw torque of approximately 10 N·m·cm^−3^.

#### 2.2.2. Filament Production

The 20 wt% obsidian PLA masterbatch was converted into filament using a single-screw extruder. To obtain the final filament compositions, the masterbatch was diluted with neat PLA to produce filaments containing 5 and 10 wt% obsidian. The four-zone temperature profile of the single-screw extruder was set to 175, 191, 200, and 204 °C. After extrusion, the filament was first passed through a 5 m long hot-water bath at 43 °C to ensure diameter stability and reduce internal stresses, and then through a ~2 m long cold-water bath at ambient temperature for final cooling. Residual surface moisture was removed using high-pressure air to improve diameter measurement accuracy. The filament diameter was continuously monitored using a laser-readout diameter sensor, and the filament was wound onto reels using an automatic winding system. Filament production was carried out by Filameon (Kayseri, Türkiye).

#### 2.2.3. FDM Printing

All specimens were printed on a Bambu Lab X1-Carbon Combo FFF printer fitted with a 0.8 mm nozzle, using the in-house produced filaments (1.75 ± 0.05 mm, laser-monitored during production). Printing used a nozzle temperature of 220 °C, a bed temperature of 60 °C, a layer height of 0.2 mm, a print speed of 50 mm s^−1^, and 100% infill with a rectilinear (±45° alternating) pattern, in a flat (horizontal) build orientation. Identical parameters were applied to all compositions.

### 2.3. Tensile Test (ASTM D638)

**Figure 2:**
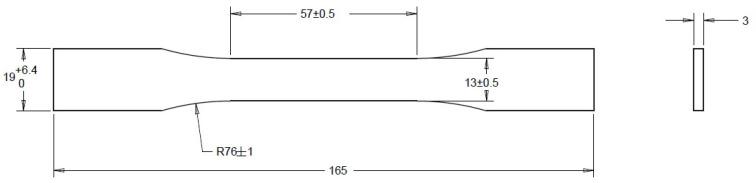
ASTM D638 Type I tensile specimen geometry.

Tensile specimens followed the ASTM D638 Type I [11] geometry with a uniform gauge cross-section of nominal area *A* = 39 mm^2^ (Figure 2). Testing was conducted on a universal testing machine at 23 ± 2 °C and 50 ± 5% RH at a constant crosshead speed of 5 mm/min. Engineering stress was calculated as follows:

*σ* = *F*/*A* (1)

where *F* is the instantaneous load. Engineering strain was calculated from the machine displacement *δ_m_* as *ε* = *δ_m_*/*L*_0_ with *L*_0_ = 50.0 mm. To bypass machine-compliance, seating, and grip-slippage artefacts, optical strain was independently obtained using Tracker v6.3.4 video analysis software by tracking two contrast markers placed 25 mm above and below the specimen centre (total span 50.0 mm) at 50 fps. Young’s modulus E was extracted per group via linear least-squares fitting in MATLAB R2023b of pooled Tracker-derived *σ*–*ε* data over the window *σ* ∈ [10, 30] MPa, which sits well within the linear-elastic regime and above the noise floor of the optical tracking (~0.02% strain, ~1–2 MPa). Seven specimens were tested per composition (n = 7).

### 2.4. Three-Point Flexure (ASTM D790) [12]

Flexural specimens were rectangular bars of length 165 mm, width *b* = 12.7 mm, and depth *h* = 8.0 mm. Three-point flexure was performed at a support span *L* = 120.0 mm and a crosshead speed of 1.28 mm/min. Maximum flexural stress, strain, and modulus were computed as follows:

(2) $$\sigma_{f}=\frac{3FL}{2bh^{2}}=0.2215\;F$$

(3) $$\varepsilon_{f}=\frac{6\delta h}{L^{2}}=3.33\times 10^{-3}\;\delta$$

(4) $$E_{f}=\frac{L^{3}k}{4bh^{3}}=66.44\;k$$

where, when *F* is expressed in N, the calculated stress is obtained in MPa; when *δ* is expressed in mm, the calculated strain is dimensionless and reported as a percentage; and when the initial load–displacement slope *k* is expressed in N/mm, the calculated modulus is obtained in MPa. Five specimens were tested per composition (n = 5).

### 2.5. Notched Charpy Impact (ASTM D6110) [13]

**Figure 3:**
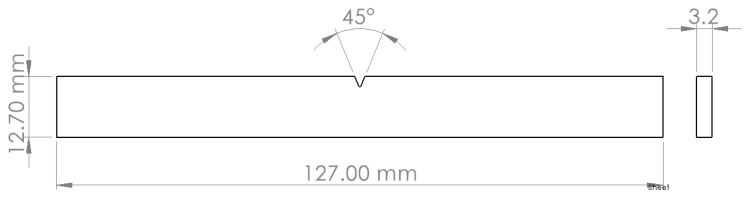
ASTM D6110 notched Charpy impact specimen geometry with 45° V-notch (R = 0.25 mm tip radius, 2.54 mm depth).

Specimens were 127 × 12.7 × 3.2 mm bars with a 45° V-notch of R = 0.25 mm tip radius and 2.54 mm depth milled at the mid-span (Figure 3). Pendulum impact testing was conducted at 23 ± 2 °C. Ten specimens were tested per composition. The energy absorbed for specimen fracture was obtained from the difference in the pendulum’s pre- and post-swing heights:

*E* = *m*·*g*·(*h*_0_ − *h*_1_) (5)

where *m* is the pendulum mass, *g* is gravitational acceleration, and *h*_0_ and *h*_1_ are the initial and post-swing heights, respectively. The notched Charpy impact strength (specific impact strength) is calculated by normalizing the absorbed energy to the remaining cross-sectional area at the notch plane:

*a_k_* = *E*/(*b*·*t*) (6)

where *b* = *W* − notch depth = 12.7 − 2.54 = 10.16 mm is the remaining ligament width and *t* = 3.2 mm is the specimen thickness. In this study, the impact energy absorbed by each specimen was read directly from the instrument in joules, and the corresponding notched Charpy impact strength (*a_k_*) was additionally calculated as the absorbed energy per unit cross-sectional area behind the notch using Equation (6); the results are therefore reported both as absorbed energy (J) and as area-normalized impact strength (kJ·m^−2^).

### 2.6. Hardness Measurement

Hardness measurements were performed in accordance with ASTM D2240 [14] using a digital Shore D durometer (Loyka, İzmir, Türkiye). Three specimens were used per composition, and three measurements were taken from different regions of each specimen, yielding nine measurements per composition (n = 9). The durometer was applied perpendicular to the specimen surface, and readings were taken after a 15 s hold.

### 2.7. Density

Cube specimens of 25 × 25 × 10 mm were printed at 100% infill (nominal volume V = 6.25 cm^3^). Mass m was determined gravimetrically on an analytical balance. Density was computed using the Archimedes method, where specimens were weighed in air ($m_{air}$) and then fully submerged in pure water ($m_{water}$):

(7) $$\rho =[m_{air}/(m_{air}-m_{airwater})]\cdot \rho_{water}$$

where $\rho_{water}$ = 1.00 g/cm^3^ at 23 °C. Measurements were made at 0.001 g accuracy. Six specimens were measured per composition (n = 6).

### 2.8. Simultaneous TGA/DSC

Simultaneous thermogravimetric and differential thermal analysis (TG/DTA) was performed using an EXSTAR 7300 TG/DTG/DTA thermal analyzer (SII NanoTechnology Inc., Chiba, Japan). Specimens of neat PLA (7.521 mg) and of the 5 and 10 wt% composites (10.491 and 13.313 mg, respectively) were placed in alumina crucibles with a matched empty crucible as reference and heated from 30 to 800 °C at 10 °C min^−1^ under flowing nitrogen, with an isothermal hold at 800 °C. The neat-PLA control was measured on the same instrument under the identical heating programme. Calorimetric transitions of neat PLA were additionally obtained by DSC (2.600 mg specimen, aluminum pan, 10 °C min^−1^, nitrogen).

### 2.9. Scanning Electron Microscopy (SEM)

Representative tensile fracture surfaces of specimens from each composition were sputter-coated with a thin gold layer and imaged using a FEI Quanta FEG 250 field-emission scanning electron microscope (FEI, Eindhoven, The Netherlands) at accelerating voltages of 10–15 kV. Imaging covered magnifications of 100×, 250×, 500×, and 1000× to evaluate the distribution of obsidian particles within the PLA matrix, the matrix–filler interface morphology, and the fracture mechanisms (brittle fracture, ductile fracture, particle pull-out, void formation).

Energy-dispersive X-ray spectroscopy (EDS; Oxford Instruments detector, 10 kV) was performed in map (area) mode on neat PLA and on the composite surfaces to determine the elemental composition and the spatial distribution of the filler. Specimens were Au sputter-coated, and the Au Mα coating peak was excluded from quantification.

Quantitative fractography was performed on the matched-magnification fracture-surface micrographs by image analysis: resolved micro-cavities were segmented after background flattening, and their areal number density and equivalent-circle size were measured.

### 2.10. Statistical Analysis

One-way analysis of variance (ANOVA) was used for the statistical evaluation of mechanical, physical, and thermal test results. ANOVA was applied to determine whether the mean values among different compositions exhibited statistically significant differences. The significance level was set at *p* < 0.05. When ANOVA indicated significant differences, post hoc tests (Bonferroni-corrected pairwise comparisons) were applied. All statistical analyses were performed using appropriate statistical software.

## 3. Results

### 3.1. Material Characterization

**Figure 4:**
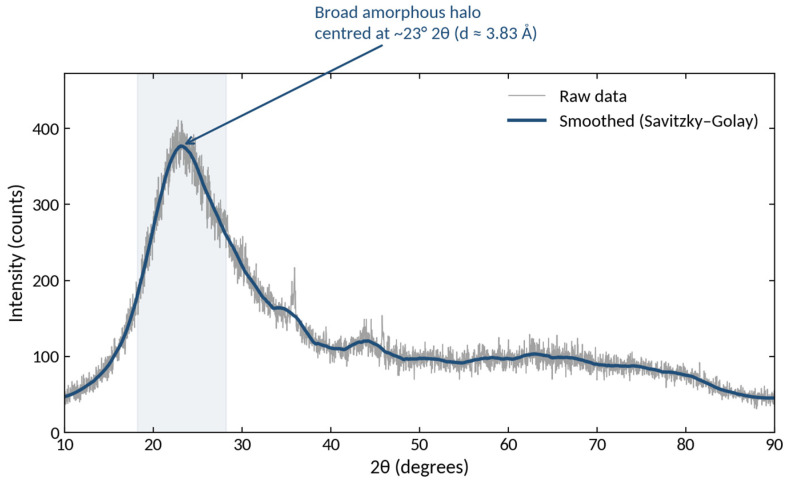
X-ray diffraction pattern of the milled Kars-region obsidian powder (Cu Kα). The single broad amorphous halo centred at ~23° 2θ and the absence of sharp crystalline reflections confirm the fully amorphous (volcanic-glass) nature of the filler.

The X-ray diffraction pattern of the milled obsidian powder (Figure 4) is dominated by a single broad, diffuse scattering halo centred at approximately 23° 2θ (mean interatomic spacing d ≈ 3.83 Å) with a full width at half-maximum of about 10°. Only weak, equally diffuse secondary modulations are discernible at higher angles, and no sharp crystalline reflections appear anywhere in the 10–90° range; automated phase identification against the powder-diffraction database returned no matching crystalline phase. This broad halo is the characteristic signature of the short-range order of an amorphous silicate network and confirms that the Kars-region obsidian is a fully amorphous volcanic glass with no detectable crystalline content. The amorphous structure is consistent with the rapid quenching of silica-rich magma that defines obsidian, and is advantageous for a polymer filler since it precludes crystalline polymorphic transitions within the PLA processing window and provides an isotropic glassy reinforcing phase.

**Figure 5:**
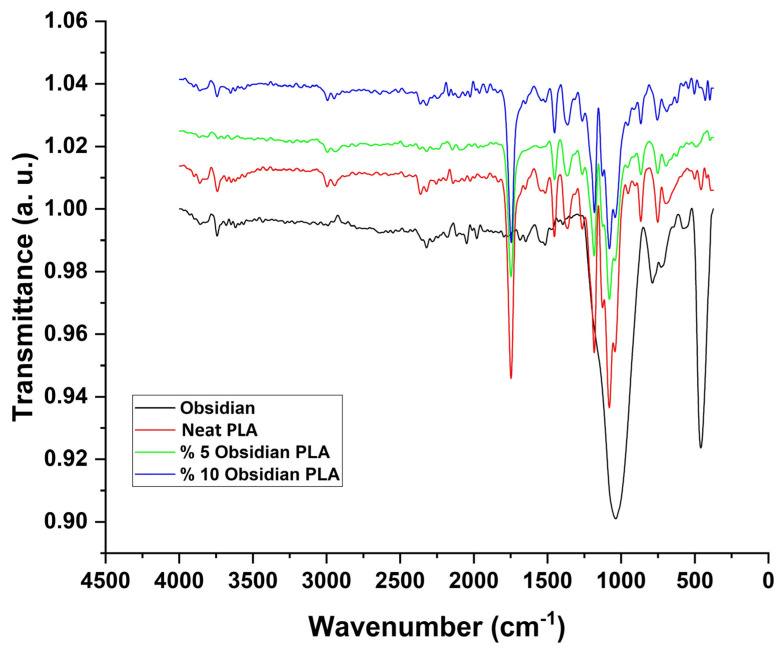
FTIR spectra (4000−400 cm^−1^) of the obsidian powder, neat PLA, and the PLA/5 wt% and PLA/10 wt% obsidian composites. The PLA ester bands (C=O ≈ 1748; C–O–C ≈ 1080 −1180 cm^−1^) are retained without shift in the composites, while the obsidian Si–O–Si bands (≈1000−1050 and ≈460 cm^−1^) are superimposed and intensify with filler content, indicating a physical (non-covalent) PLA–obsidian interaction.

FTIR spectra of the neat PLA, the 5 and 10 wt% composites, and the obsidian powder are compared in Figure 5. The neat-PLA spectrum displays all the characteristic ester bands of PLA: the strong C=O stretching band at ≈1748 cm^−1^, the C–O–C asymmetric and symmetric stretching bands at ≈1180, 1128 and 1080 cm^−1^, the CH_3_ deformation bands at ≈1452 and ≈1382 cm^−1^, and the C–H stretching doublet at ≈2995 and ≈2945 cm^−1^. The obsidian spectrum is dominated by the Si–O–Si asymmetric stretching envelope centred near ≈1000–1050 cm^−1^ and the Si–O–Si bending band near ≈460 cm^−1^, together with a weak broad O–H/adsorbed-water feature near 3400 cm^−1^, confirming the silicate-glass nature of the filler. The composite spectra reproduce the full set of PLA bands at unchanged positions, with the silicate Si–O contribution superimposed on the PLA C–O–C envelope in the 1000–1100 cm^−1^ region and growing slightly with obsidian content. Crucially, the position and shape of the PLA carbonyl band at 1748 cm^−1^ remain unaltered upon obsidian incorporation, and no new bands appear. The absence of any carbonyl shift, band splitting, or new absorption indicates that no covalent or strong specific bonds form between PLA and the unmodified obsidian surface; the PLA–obsidian interaction is therefore essentially physical (van der Waals and weak hydrogen bonding). This spectroscopic result independently corroborates the weak interfacial coupling inferred from the SEM debonding features (Section 3.8) and the modulus–strength decoupling (Section 4.2), and it provides the chemical rationale for the silane-functionalization strategy proposed as future work.

**Figure 6:**
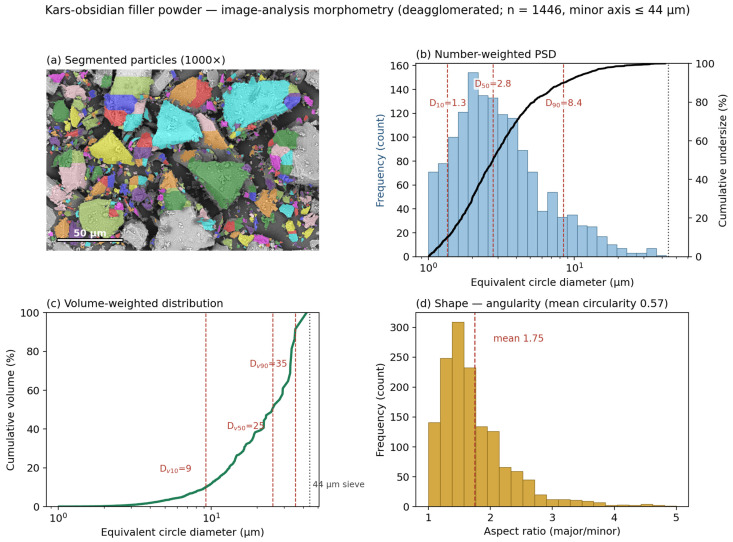
Image analysis morphometry of the milled Kars obsidian powder (deagglomerated; n = 1446, minor axis ≤ 44 µm): (**a**) segmented particles (1000×, scale bar 50 µm); (**b**) number-weighted PSD with cumulative undersize (D10 = 1.3, D50 = 2.8, D90 = 8.4 µm); (**c**) volume-weighted cumulative distribution (Dv10 = 9, Dv50 = 25, Dv90 = 35 µm); (**d**) aspect ratio distribution (mean 1.75; mean circularity 0.57).

The milled obsidian powder consists of angular, sharp-edged fragments bounded by conchoidal fracture surfaces characteristic of mechanically comminuted volcanic glass, with no distinct secondary phase (Figure 1b). Image analysis morphometry of the deagglomerated particle population (Figure 6) gives volume-weighted distributions—the quantity directly comparable to laser-diffraction sizing—of =9 µm, =25 µm, and =35 µm, all below the 44 µm sieve aperture. The number-weighted distribution (D10 = 1.3 µm, D50 = 2.8 µm, D90 = 8.4 µm) is dominated by a large population of fine micron-scale splinters generated by the ball-milling of brittle glass. The particles are markedly angular, with a mean circularity of 0.57 and a mean aspect ratio of 1.75. This broad, fine–rich, angular morphology is mechanically relevant: the abundant fine fraction generates a high particle–matrix interfacial area and hence many potential debonding sites under load, while the coarsest fragments act as the local stress concentrators identified in the fractographic analysis (Section 3.8).

**Figure 7:**
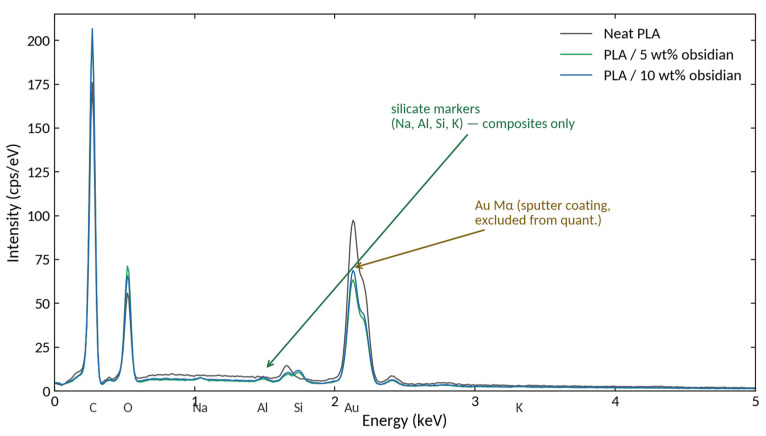
EDS map-sum spectra of neat PLA and the PLA/5 wt% and PLA/10 wt% obsidian composites (10 kV). Neat PLA shows only C and O; the composites additionally display the obsidian silicate markers (Na, Al, Si, K). The Au Mα peak (~2.12 keV) is from the sputter coating and is excluded from quantification.

**Figure 8:**
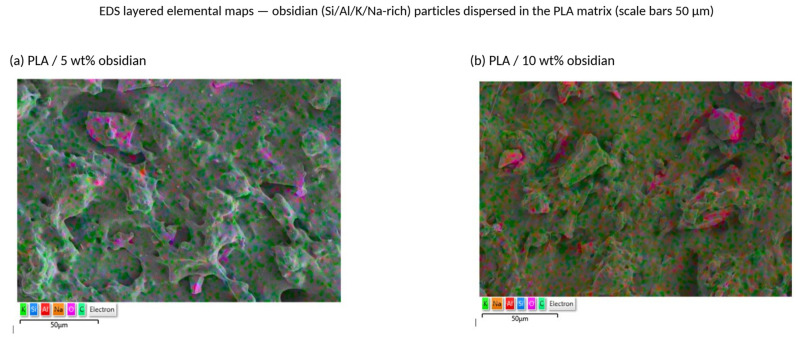
EDS layered elemental maps of the (**a**) 5 wt% and (**b**) 10 wt% composites, showing obsidian (Si/Al/K/Na-rich) particles dispersed across the PLA (C-rich) matrix; particle density increases with loading. Scale bars: 50 µm.

EDS map-sum spectra (Figure 7) and the corresponding quantification (Table 1) confirm the incorporation and distribution of the obsidian filler. Neat PLA shows only carbon and oxygen (C 69.6, O 30.4 wt%), consistent with the polyester structure. Both composites additionally exhibit the silicate markers of the obsidian—Si, Al, Na and K—in proportions that increase with filler loading (Si 1.4 and 1.6 wt%, Al 0.4 and 0.5 wt% at 5 and 10 wt%, respectively), matching the rhyolitic (high-silica) composition of the glass. Because the spectra are summed over a mapped area, the consistent detection of these elements indicates that the filler is distributed across the matrix rather than confined to isolated regions. The Au Mα line at ~2.12 keV originates from the conductive sputter coating and is excluded from quantification. Elemental maps (Figure 8) localize the Si and Al signals in discrete micron-scale particles dispersed across the carbon-rich matrix, with occasional clusters—directly confirming the spatial distribution of the obsidian and revealing a mild tendency to agglomerate at higher loading; the corresponding neat-PLA maps show only carbon and oxygen.

### 3.2. Tensile Behaviour

**Figure 9:**
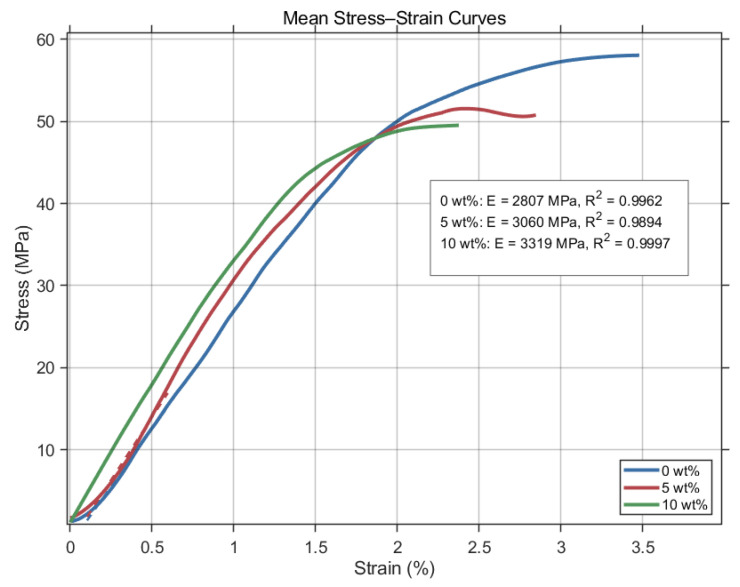
Engineering stress–strain curves of FDM-printed PLA/Kars obsidian composites under tensile loading at the three compositions.

The tensile response of all three compositions was approximately linear up to ~25–30 MPa, followed by a short non-linear yielding region and brittle fracture without a pronounced post-peak plateau; this is consistent with the well-documented brittle character of FDM-printed PLA [15,16]. Representative engineering stress–strain curves are shown in Figure 9. Group means and standard deviations are summarized in Table 2.

Adding 5 wt% obsidian reduced it by 12.4% relative to neat PLA, while 10 wt% reduced it by an additional 5.0% (cumulative −17.3%). The strain at peak load decreased monotonically from 10.32% (neat) to 8.67% (5 wt%) and 8.19% (10 wt%). In sharp contrast to the strength and ductility decrease, Young’s modulus rose monotonically by 9.0% at 5 wt% and 18.2% at 10 wt%; all per-group linear fits exhibited R^2^ > 0.989, indicating an excellent linear regime within the selected fitting window.

One-way ANOVA on returned F(2, 18) = 329, *p* ≈ 7 × 10^−15^. Bonferroni-corrected post hoc pairwise comparisons were all highly significant: 0 vs. 5 wt% (Δ = −7.12 MPa, *p* < 10^−4^), 0 vs. 10 wt% (Δ = −9.99 MPa, *p* < 10^−4^), and 5 vs. 10 wt% (Δ = −2.87 MPa, *p* < 10^−4^). For *ε* at peak, ANOVA gave F(2, 18) = 56.2, *p* ≈ 2 × 10^−8^; the 0 vs. 5 wt% and 0 vs. 10 wt% contrasts were significant, while the 5 vs. 10 wt% contrast was not significant after Bonferroni correction (*p* = 0.17), indicating that most of the ductility loss occurred between 0 and 5 wt%, with little additional loss at higher loading.

### 3.3. Flexural Behaviour

**Figure 10:**
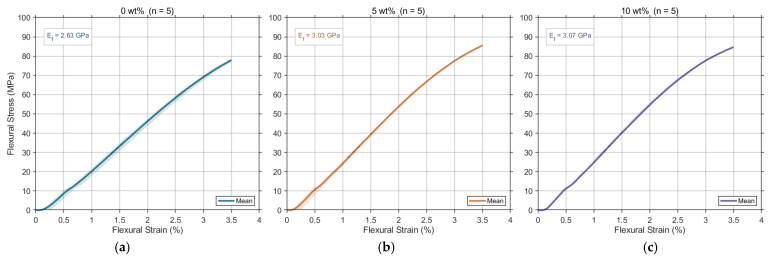
Mean flexural stress–strain curves of FDM-printed PLA/-obsidian composites at (**a**) 0 wt%, (**b**) 5 wt%, and (**c**) 10 wt% obsidian loading. Solid lines indicate group-mean curves obtained by interpolating all valid specimens onto a common flexural-strain axis followed by Savitzky–Golay smoothing (order 3, frame 121). Shaded bands indicate the min–max distribution among replicates. Dashed lines indicate the algorithmically selected linear-elastic window used for the per-group flexural-modulus fit (R^2^ ≥ 0.97 for all groups).

Three-point flexure was performed per ASTM D790. For each composition, F–δ traces of all valid specimens were interpolated onto a common flexural-strain axis, the mean curve was obtained using a Savitzky–Golay filter (order 3, frame 121), and the linear-elastic window was selected algorithmically as the continuous segment within the 5–45% peak-load range that maximized the coefficient of determination of a positive-slope linear fit. A single representative value was reported per group from this pooled fit. The mean flexural stress–strain curves with min–max distribution bands are shown in Figure 10.

All three compositions exhibited the expected three-stage response of a glassy thermoplastic under flexure: an essentially linear-elastic regime up to ≈ 0.5%, a non-linear pre-peak region, and a gradual load decrease near peak without sharp catastrophic fracture within the analyzed strain window. Per-specimen peak values are summarized in Table 3. Flexural strength remained essentially unchanged between neat PLA and the 5 wt% composite (92.07 → 92.18 MPa) and decreased by only 3.5% at 10 wt% (88.84 MPa). This is a striking contrast to the monotonic 17.3% decrease in tensile strength over the same composition range and indicates that flexural-mode loading is markedly more tolerant of obsidian filler than axial tension.

The flexural strain at peak load followed the tensile-ductility trend, decreasing monotonically from 5.09% (neat) to 4.29% at 5 wt% and 4.07% at 10 wt%. The mid-span displacement at peak *δ*_peak correspondingly dropped from 15.27 to 12.20 mm (−20.1%).

The flexural modulus increased monotonically with obsidian content (Figure 10, Table 3; n = 5 per group): neat PLA, =2.63 GPa (R^2^ = 0.978); 5 wt% obsidian, =3.03 GPa (R^2^ = 0.972, +15.2% vs. neat PLA); 10 wt% obsidian, =3.07 GPa (R^2^ = 0.981, +16.7% vs. neat PLA).

Two features of the data warrant comment. First, the increment between 5 and 10 wt% is small (+1.3%, from 3.03 to 3.07 GPa); this contrasts sharply with the +8.5% rise observed for the tensile modulus over the same composition step (3.06 → 3.32 GPa, Table 2). This early saturation in flexural stiffness alongside continued tensile modulus gain is consistent with SEM evidence (Section 3.8) of localized agglomerates and micro-void clusters at 10 wt% that preferentially distort the outer-fibre flexural response—where flexural stress is concentrated—but do not significantly affect the bulk axial stiffness sampled by tensile testing. Second, flexural modulus values are systematically 6–8% lower than the corresponding tensile modulus values (e.g., 2.63 vs. 2.81 GPa for neat PLA)—a difference well documented in the FDM-printed PLA literature, attributed to limited shear transfer between deposited beads via interlayer bonding during flexure.

### 3.4. Notched Charpy Impact

Notched Charpy energies are listed in Table 4. Group means were 0.640 ± 0.178 J for neat PLA, 0.562 ± 0.048 J at 5 wt%, and 0.625 ± 0.411 J at 10 wt%. Expressed as area-normalized impact strength behind the notch (Equation (6); reference ligament area *b*·*t* = 32.51 mm^2^), these means correspond to a_k_ = 19.7 ± 5.5, 17.3 ± 1.5 and 19.2 ± 12.6 kJ·m^−2^ for the neat PLA, 5 wt% and 10 wt% specimens, respectively (Table 4); area normalization preserves the same ranking and the same statistically indistinguishable trend already evident in the absorbed-energy data. The 5 wt% group showed a small (12%) decrease relative to the control. The 10 wt% mean is statistically indistinguishable from neat PLA; however, the intra-group standard deviation was 0.411 J—indicating a variance source comparable to the mean itself. Individual measurements ranged from 0.52 to 1.00 J, and this wide distribution implies that fracture initiation in the 10 wt% formulation exhibits strong specimen-to-specimen heterogeneity: individual fractures are sensitive to the stochastic location of sample-specific defects such as local particle agglomeration, micro-void distribution, or inter-bead boundary quality.

The large intra-group standard deviation of the 10 wt% Charpy impact strength (0.411 J, comparable to its mean) is attributed to the stochastic distribution of particle agglomerates and inter-bead microvoids that act as fracture-initiation sites; this scatter is itself a signature of inhomogeneous dispersion at the higher loading.

### 3.5. Shore D Hardness

Shore D hardness, summarized in Table 5, rose from 80.22 ± 0.94 in neat PLA to 82.33 ± 1.17 at 5 wt% and 82.44 ± 0.92 at 10 wt%. The increase between 0 and 5 wt% (Δ = +2.11 points) is larger than that between 5 and 10 wt% (Δ = +0.11), consistent with a saturating contribution of the harder ceramic phase to the indentation response in a regime in which the indenter footprint averages over multiple particles.

### 3.6. Density

The density values (Table 5) rose from 1.2283 ± 0.0010 g/cm^3^ in neat PLA to 1.2291 ± 0.0017 at 5 wt% and 1.2343 ± 0.0015 at 10 wt%. Assuming an obsidian literature density of 2.35 g/cm^3^ and a perfect rule of mixtures with no void contribution, the predicted composite densities would be approximately 1.273 g/cm^3^ at 5 wt% and 1.320 g/cm^3^ at 10 wt%. The measured values lie below these predictions; the deficit is consistent with the formation of micro-voids and inter-bead porosity during FDM printing, well known to lower printed-part density relative to filament density [15,16,17].

### 3.7. Thermogravimetric and Differential Thermal Analysis

**Figure 11:**
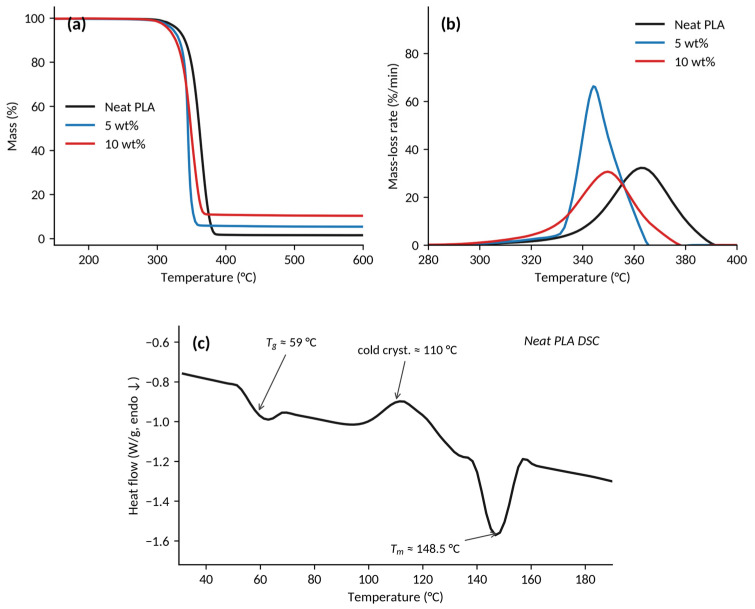
Thermal analysis of neat PLA and the FDM-printed PLA/Kars obsidian composites (5 and 10 wt%): (**a**) TGA mass-loss curves; (**b**) corresponding DTG (mass-loss rate) curves; (**c**) DSC thermogram of neat PLA, showing the glass transition (≈59 °C), the cold-crystallization exotherm (≈110 °C), and the melting endotherm (≈148.5 °C). All TGA/DTG measurements were acquired from 30 to 800 °C at 10 °C min^−1^ under flowing N_2_ on the same instrument.

The TGA and corresponding DTG curves of neat PLA and the 5 and 10 wt% composites are shown in Figure 11. All three compositions exhibited the single, sharp degradation step characteristic of PLA, with decomposition essentially complete by ~400 °C. Referenced to neat PLA, obsidian systematically advances the onset of decomposition. The 5% mass-loss temperature (T_5_) decreased from 331.1 °C for neat PLA to 322.3 °C at 5 wt% and 316.5 °C at 10 wt%, and the 10% mass-loss temperature (T_10_) from 341.2 to 332.1 and 326.9 °C, respectively (Table 6)—a monotonic reduction of ~9 °C at 5 wt% and ~15 °C at 10 wt%, with a further ~5–6 °C drop between 5 and 10 wt%. The temperature of maximum decomposition rate was 345.8 °C at 5 wt% and 349.8 °C at 10 wt%.

The most pronounced difference in the DTG data lies in the rate and width of the main decomposition event. The normalized maximum decomposition rate fell from ~49.6%/min at 5 wt% to ~26.9%/min at 10 wt% (~46% lower), and the T_20_–T_80_ decomposition interval broadened from 9.7 to 23.5 °C. The high-temperature residue increased from ~1.5 wt% for neat PLA to ~5.41 wt% at 5 wt% and ~10.35 wt% at 10 wt%; the composite residues closely match the nominal obsidian loadings, confirming that the filler is thermally stable within the TGA window and providing an independent cross-check of filler content. Thus, although obsidian addition does not raise the early-decomposition temperature, it reduces the peak decomposition rate, broadens the decomposition event, and increases the high-temperature residue.

Calorimetric transitions were obtained from a dedicated DSC scan of neat PLA: a glass transition at ~58–60 °C, a cold-crystallization exotherm at ~110 °C, and a melting endotherm at ~148.5 °C, corresponding to a degree of crystallinity of ~9% (ΔH°m = 93.7 J g^−1^) and typical of the largely amorphous as-printed state of rapidly cooled FDM-PLA. In the simultaneous DTA signal of the composites, the PLA melting endotherm remained essentially unchanged (~147.8 °C at 5 wt%, ~149.3 °C at 10 wt%), indicating that obsidian neither acts as a nucleating agent nor alters the PLA crystal structure at the loadings examined.

### 3.8. Fractographic Analysis

**Figure 12:**
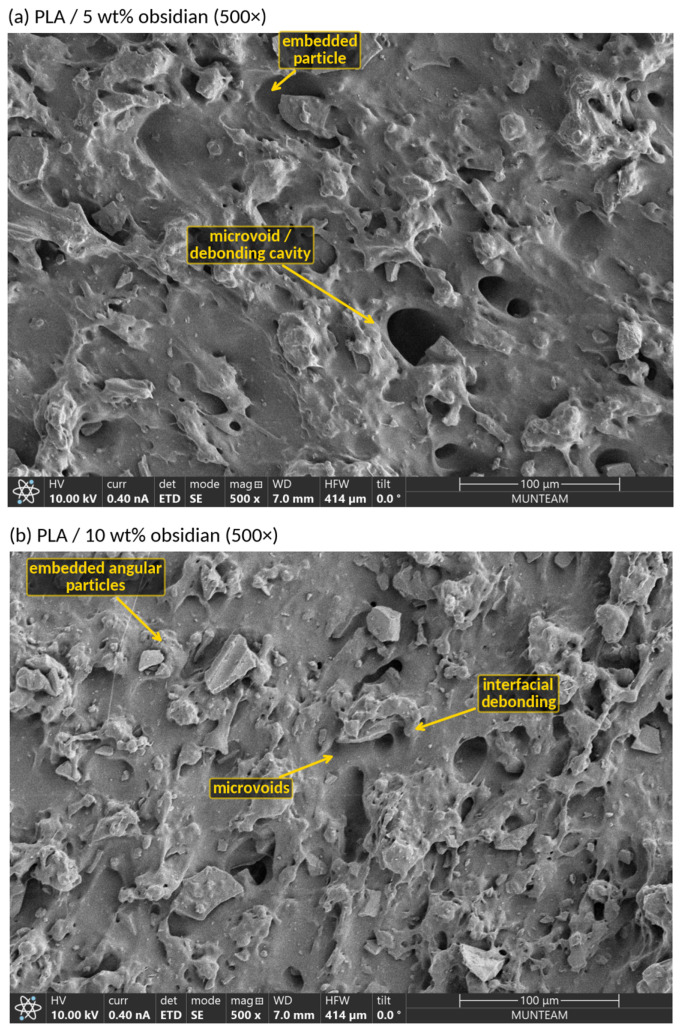
Tensile fracture surfaces (SE, 500×) of the (**a**) 5 wt% and (**b**) 10 wt% obsidian composites, annotated for embedded angular particles, interfacial debonding cavities, and microvoids. The density of embedded particles and debonding cavities increases with loading. Scale bars: 100 µm.

To complement the qualitative fractography, the matched 500× tensile fracture surfaces of the 5 and 10 wt% composites (Figure 12) were analyzed quantitatively. Both display the same feature set—embedded angular obsidian particles, interfacial debonding cavities, microvoids and localized matrix tearing—but their abundance increases with loading: the areal density of resolved micro-cavities rises roughly two-fold, from ~510 mm^−2^ at 5 wt% to ~1100 mm^−2^ at 10 wt% (500×; a comparable increase at 1000×), while the mean cavity diameter is essentially unchanged (~2–3 µm). Higher obsidian content therefore multiplies the number of interfacial debonding sites rather than enlarging them. The automated void-area fraction is not reliable on secondary-electron fracture topography owing to shadowing; the bulk void content is quantified independently by the Archimedes apparent porosity, which rises from ~1% to ~5% over the same range (Section 3.6). This increasing population of weakly bonded particles and associated microvoids provides the fractographic basis for the simultaneous reduction in tensile and impact properties and for the larger Charpy scatter at 10 wt%.

#### 3.8.1. Macroscopic Post-Fracture Appearance

**Figure 13:**
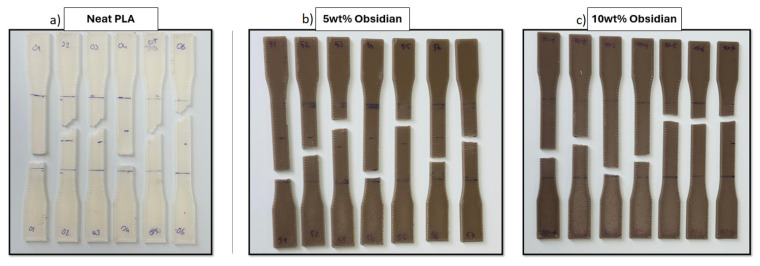
Macroscopic post-fracture appearance of tensile specimens: (**a**) neat PLA; (**b**) PLA/5 wt% obsidian; (**c**) PLA/10 wt% obsidian.

Before discussing the micro-scale fracture features revealed by SEM, the macroscopic post-fracture appearance of the tensile specimens provides direct visual evidence of the composition-dependent change in damage character. Figure 13 presents representative post-fracture photographs of fractured tensile specimens for the three compositions; each specimen is shown as upper and lower halves after separation.

Three observations are immediately apparent. (i) Colour progression: Macroscopic specimen colour varied continuously from translucent ivory in neat PLA (Figure 13a) to light brown at 5 wt% obsidian (Figure 13b) and to a darker, more saturated brown at 10 wt% (Figure 13c). This colour gradient follows the optical rule of mixtures and visually confirms the successful incorporation and homogeneous spatial distribution of obsidian filler at the macroscopic scale; no streaks, agglomerated dark spots, or colour heterogeneities appear within the gauge length of any specimen. (ii) Fracture-edge character: The most diagnostic macroscopic feature is the geometry of the fracture edges themselves. In neat-PLA specimens (Figure 13a), the fractured ends frequently exhibit shallow, feather-like, torn edges with visible fibrillar extensions—macroscopic evidence of plastic drawing before final separation. In the 5 wt% composites (Figure 13b), the fracture edges become noticeably cleaner and more linear but retain some surface roughness and small angular protrusions. At 10 wt% (Figure 13c), the fracture is consistently sharp and nearly perfectly perpendicular to the loading axis, with smooth, clean edges diagnostic of brittle separation. (iii) Fracture location: In all three compositions, fracture consistently occurred within the gauge-length section, confirming that the recorded tensile strengths reflect intrinsic material behaviour rather than stress-concentration artefacts associated with the dogbone geometry.

Together, these macroscopic observations independently support the same ductile → transitional → brittle progression that is verified at the micro-scale by the SEM analysis presented in Section 3.8.2, Section 3.8.3, Section 3.8.4 and Section 3.8.5.

**Figure 14:**
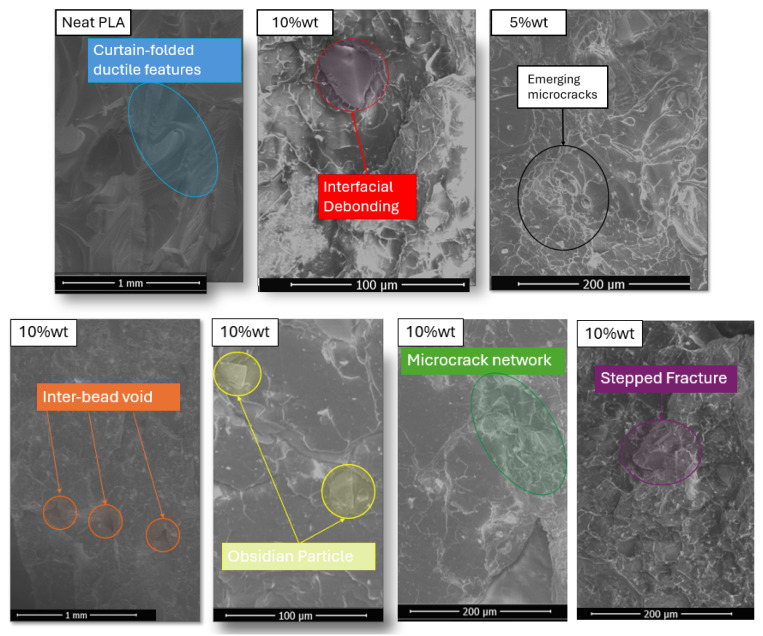
SEM micrographs of tensile fracture surfaces of FDM-printed PLA/Kars obsidian composites at three compositions (neat PLA, 5 wt%, 10 wt%) and four magnifications (100×, 250×, 500×, 1000×). Each micrograph is annotated in-image with its composition and magnification.

The tensile fracture surfaces of the three compositions—neat PLA, PLA/5 wt% obsidian, and PLA/10 wt% obsidian—were examined by scanning electron microscopy to identify the dominant damage mechanisms governing the mechanical–property gradient reported in Section 3.1, Section 3.2 and Section 3.3. Three independent specimens for each composition were analyzed at magnifications of 100×, 250×, 500×, and 1000×. Representative micrographs are compiled in Figure 14, where each micrograph is annotated in-image with its composition and magnification, allowing the composition-dependent morphological progression to be read directly from the figure.

#### 3.8.2. Neat PLA—Ductile Fracture Morphology

At low magnification, the fracture surface of neat PLA (Figure 14) exhibits an unusual morphology dominated by smooth, large-scale, curtain-like folds with deep dark grooves between adjacent fold sets. Periodic FDM inter-bead voids are not visible at this scale because the matrix has undergone sufficient local plastic deformation during tensile loading to obscure the original print geometry. This observation alone indicates that the FDM-printed PLA used in this study exhibits notable ductility, consistent with the relatively high elongation at peak load reported for the 0 wt% group (*ε* = 10.32 ± 0.33%; Table 2).

At high magnification (Figure 14), the ductile character of the neat-PLA fracture surface is decisively confirmed by the presence of pronounced fibrillar drawing features. The fracture surface exhibits dramatic feather-like fibrillation in which polymer chains have been drawn in the direction of the applied load, producing long, parallel, smooth fibrils that extend radially outward from a central feature. These features are the classical secondary-electron signature of cold-drawing of an amorphous thermoplastic above its glass-transition temperature under tensile stress: local necking, chain orientation, and partial strain-hardening before final separation. The complete absence of microcracks, particles, or stepped fracture facets confirms that neat FDM-printed PLA fractures in a quasi-ductile manner in the present specimens—despite its known intrinsic brittleness in injection-moulded form—and that significant plastic dissipation occurs during fracture.

#### 3.8.3. PLA/5 wt% Obsidian—Transitional Morphology

At low magnification, the 5 wt% composite (Figure 14) exhibits a fracture surface markedly different from neat PLA: the large-scale curtain folds have been largely suppressed, replaced by a coarser, rougher topography containing embedded angular features. This change reflects the partial loss of plastic-flow capacity upon incorporation of the rigid obsidian filler: the polymer can no longer fully flow around dispersed particles, and the surface texture begins to reflect the underlying particle distribution rather than the deposition-flow geometry. The periodic FDM bead structure remains visible but is less prominently separated than at 10 wt% loading.

At high magnification (Figure 14), the 5 wt% composite exhibits a clearly transitional morphology that combines features inherited from both end-member compositions. Regions of residual matrix flow coexist with emerging microcracks that appear as fine bright lines distributed within the matrix. Some angular obsidian particles can be discerned, but are not as visually dominant as at 10 wt%. The simultaneous presence of (i) residual plastic-flow features and (ii) incipient microcrack networks is diagnostic of the brittle–ductile transition: the matrix still retains some capacity for local deformation, but the addition of rigid inclusions has begun to nucleate crack-initiation events at particle–matrix interfaces. This morphological transition is consistent with the mechanical–property trends observed at 5 wt%: a moderate decrease in strength (from 57.6 to 50.5 MPa, −12.4%) and a corresponding decrease in ductility (*ε* from 10.32 to 8.67%, −16.0%), without yet reaching the more pronounced losses observed at 10 wt%.

#### 3.8.4. PLA/10 wt% Obsidian—Brittle Composite Fracture

At low magnification, the 10 wt% composite (Figure 14) shows a fully developed FDM bead architecture characterized by a sequence of periodic V-shaped inter-bead voids. These voids appear as triangular dark depressions aligned along the deposition direction, with spacing close to the nominal bead width. Their visibility—in contrast to neat PLA where the flow obscures the voids—indicates that the matrix at 10 wt% obsidian loading has lost the local post-fracture deformation capacity that would otherwise smooth the underlying bead geometry. The voids constitute a discrete population of pre-existing stress concentrators that act as preferred crack-initiation sites under tensile loading, and they also contribute to the residual porosity, explaining the rule-of-mixtures density deficit reported in Section 3.6.

At high magnification (Figure 14), three damage mechanisms appear simultaneously. Obsidian particles appear as angular, sharp-edged inclusions exhibiting the conchoidal fracture surfaces characteristic of mechanically milled volcanic glass; particle sizes remain well within the 44 µm sieve limit used during preparation. A dense microcrack network is observed in regions of high local stress concentration, with bright, interconnected linear features interpreted as the secondary-electron signature of crack edges raised slightly above the surrounding matrix plane. Finally, a long interfacial debonding line surrounding a larger particle is observed, indicating that the polymer matrix has separated from the silicate surface along this contour during crack propagation. The continuity and brightness of this debonding line are diagnostic of weak interfacial bonding: under tensile loading, the load that should be transferred from matrix to rigid filler is instead released at the interface. This observation is consistent with a polar–apolar mismatch between PLA (a polyester carrying carbonyl and hydroxyl end groups) and the unmodified silicate surface of obsidian, which lacks the reactive functional groups required to form covalent or strong hydrogen bonds with the matrix.

#### 3.8.5. Composition-Dependent Morphological Progression

Figure 14 reveals a clear and continuous morphological progression with increasing obsidian content. Neat PLA exhibits predominantly ductile fracture: smooth curtain folds at low magnification, prominent fibrillar drawing at high magnification, and no discrete crack-initiation sites. PLA/5 wt% obsidian shows a transitional morphology in which residual matrix plasticity coexists with emerging microcracks. PLA/10 wt% obsidian shows a fully brittle composite fracture: sharply visible FDM bead architecture, abundant particle features, dense microcrack networks, and clear interfacial debonding around larger particles.

This progression simultaneously explains the principal mechanical observations of Section 3.1, Section 3.2 and Section 3.3. The tensile strength decreases from 57.6 to 50.5 to 47.6 MPa (−12.4% and −17.3% at 5 and 10 wt%, respectively, relative to neat PLA) because the damage mechanism shifts from cohesive matrix fracture—capable of dissipating energy via plastic flow—to interfacial debonding and microcrack coalescence, which dissipate much less energy per unit crack area. The elongation at peak load decreases from 10.32 to 8.67 to 8.19% (−16.0% and −20.6%) because the plastic-drawing capacity prominent in the fibrillar morphology of neat PLA is progressively suppressed by rigid filler inclusions. The flexural modulus increases from 2.63 to 3.03 to 3.07 GPa (+15.2 and +16.7%) because the high intrinsic modulus of obsidian particles (60–80 GPa) contributes additively in the linear-elastic regime through partial interfacial coupling, even when the interfacial bond is too weak to transfer full damage loads. The notched Charpy impact-energy distribution widens markedly at 10 wt% (SD 0.41 J on a mean of 0.62 J) because the stochastic distribution of large microcrack clusters and inter-bead-aligned pull-out regions varies appreciably between individual specimens within the small Charpy gauge volume.

#### 3.8.6. Implications and Limitations

The fractographic evidence consistently supports a single unified mechanism: the addition of obsidian filler to PLA progressively suppresses the local plastic deformation responsible for the limited ductility of the neat polymer, while at the same time exposing crack-initiation sites at the particle–matrix interfaces. The intermediate composition (5 wt%) provides a useful compromise between strength loss and stiffness gain, while the 10 wt% loading crosses the threshold at which interfacial debonding and microcrack coalescence dominate the damage process. Future studies aimed at improving filler–matrix coupling—for example, silane surface treatment of obsidian particles (e.g., 3-aminopropyltriethoxysilane, APTES) or the addition of a maleic-anhydride-grafted PLA compatibilizer—are expected to promote load transfer across the interface and recover part of the lost tensile strength by suppressing the extended debonding lines observed in Figure 14 at 10 wt% loading.

## 4. Discussion

This study has systematically characterized the mechanical, thermal, and microstructural behaviour of FDM-printed PLA/Kars obsidian composites. Considered together, the trade-offs introduced by obsidian addition—gains in modulus and hardness, losses in tensile strength and ductility, and a dual effect on thermal stability—can be read within a single unified framework: the incorporation of a rigid, chemically inert, amorphous silicate phase into a polyester matrix under conditions of limited interfacial affinity. The performance ceiling is set not by the filler itself but by interfacial quality and residual FDM-induced porosity, in line with the general pattern reported for particulate-filled MEX-PLA systems [1,2,4].

### 4.1. Modulus–Strength Trade-Off and Interfacial Coupling

The tensile experiments exhibited the modulus–strength trade-off typical of rigid-filled thermoplastics: Young’s modulus rose by +9.0% at 5 wt% and by +18.2% at 10 wt% loading, while ultimate tensile strength decreased by 12.4% and 17.3%, respectively, over the same composition range (Table 2). In the linear-elastic regime, rigid obsidian particles (intrinsic Young’s modulus ≈60–80 GPa [7,8]) contribute a rule-of-mixtures-type stiffness gain even through partial mechanical interlock, without requiring complete interfacial coupling. Near peak load, however, local stress concentrations must be transmitted across the filler–matrix interface; the absence of strong covalent or hydrogen bonding between the carbonyl and hydroxyl end groups of PLA and the unmodified silicate surface limits this load transfer and converts particle–matrix interfaces into preferential crack-initiation sites. The extended interfacial debonding lines and pull-out cavities visible in Figure 14 at 10 wt% loading provide direct visual evidence of this mechanism.

To assess whether the modulus increase reflects genuine reinforcement, the measured tensile moduli were compared with micromechanical predictions (Table 7). Using filler volume fractions of 2.70 and 5.54 vol% (for 5 and 10 wt%, from $\rho_{obsidian}$ = 2.35 and = 1.24 g/cm^3^), a matrix modulus = 2.81 GPa and a filler modulus = 70 GPa, the Voigt and Reuss rule-of-mixtures bounds were evaluated together with the Halpin–Tsai model for equiaxed particles (ξ = 2). The measured moduli (3.06 and 3.32 GPa) lie between the Reuss lower bound (2.89 and 2.97 GPa) and the Voigt upper bound (4.62 and 6.53 GPa) and are reproduced almost exactly by the Halpin–Tsai model (3.02 and 3.25 GPa). The close Halpin–Tsai agreement indicates that the stiffness gain is a genuine elastic-reinforcement effect governed by elastic load sharing—a regime far less sensitive to interfacial bond quality than strength—which explains why the modulus rises even though the weak interface simultaneously lowers the tensile strength. 

This pattern has been consistently reported for other mineral and ceramic fillers added to PLA. Efstratiadis et al. [1] observed a simultaneous modulus rise and tensile strength loss in silica/glass-bead-filled FDM-PLA; Hedjazi et al. [2] showed that for 15 vol% ceramic-reinforced PLA, stiffness is largely retained across print orientations, but weak interfacial coupling caps tensile strength performance. Vidakis et al. [5] reported a 3–6 wt% optimum window for glass-filled MEX-PLA, while Aksoy et al. [18] located the best overall mechanical balance for PLA/expanded perlite at only 2.5 wt% loading. The 17.3% tensile strength loss of the present system sits at the upper end of this literature range, indicating that the unmodified surface of Kars obsidian exhibits weaker intrinsic interfacial affinity than comparable mineral fillers (glass, hydroxyapatite, diatomaceous earth). The broader review by Çoban and Yılmaz [4] reinforces the same conclusion across the volcanic particulate–polymer composite literature.

The Shore D hardness results (Table 5) are consistent with this interpretation. The +2.11-point sharp rise from 0 to 5 wt% contrasts with the negligible +0.11-point change from 5 to 10 wt%; this saturation behaviour is compatible with an indentation regime in which the contact area averages over multiple particles, and a 5 wt% loading already supplies sufficient near-surface particle population. Further loading produces no additional hardness gain, yet the small but consistent total increase (Δ = +2.2 points) confirms that the harder ceramic phase of obsidian still contributes measurably to the indentation response. Similar saturation trends are well documented in mineral-filled PLA literature [6,17].

Benchmarking against mineral-filled PLA confirms that our modulus-up/strength-down behaviour is the rule rather than an anomaly (Table 8). Untreated particulate and fibrous fillers—glass, basalt, fly ash, perlite—consistently raise stiffness (typically +10 to +75%) while leaving tensile strength unchanged or reduced because elastic load-sharing is insensitive to interface quality, whereas strength is not. Our +18.2% modulus gain matches glass-bead PLA (+16%), but our −17.3% tensile loss lies at the upper end of comparable particulate systems, where particulate basalt and perlite report only minor reductions. This positions the unmodified, amorphous obsidian surface as a notably weak adherent—a deficit that the literature shows is recoverable: silane treatment of basalt powder/fibre and compatibilization restore load transfer, and favourably bonded fillers such as montmorillonite even raise PLA strength (+63% at 5 phr). The flexural tolerance and low (~2.5–6 wt%) optimum windows we observe are likewise consistent with these systems.

### 4.2. Tensile–Flexural Decoupling and the ≈5 wt% Optimum Window

One of the most notable findings of this study is the clear decoupling between tensile and three-point flexural moduli within the 5–10 wt% interval: the tensile modulus rose monotonically from 3.06 to 3.32 GPa (+8.5%), whereas the flexural modulus changed only by +1.3% (3.03 → 3.07 GPa) and effectively plateaued (Table 3). This decoupling can be explained by the different material volumes that the two test modes mechanically sample: tensile loading engages the entire cross-section of the dogbone specimen simultaneously and samples the bulk axial stiffness, while three-point flexure concentrates stress at the outer-fibre surface and produces a linearly decaying stress distribution towards the neutral axis. The local particle agglomerates and inter-bead micro-void clusters observed under SEM at 10 wt% (Figure 14) disproportionately distort the flexural response because they preferentially localize within the outer-fibre region where stress is concentrated. Axial tensile loading, on the other hand, dilutes the effect of agglomerates across the full cross-section, so the additive modulus gain from local stiffening is preserved. To the authors’ knowledge, this tensile/flexural scaling difference has not been explicitly emphasized in the mineral-filled MEX-PLA literature, and it represents an important finding regarding how filler-dependent defect populations shape the choice of a mechanical characterization strategy in FDM-printed composites.

The fact that flexural strength dropped by only 3.5% at 10 wt% loading (92.07 → 88.84 MPa, Table 3)—in stark contrast to the 17.3% decrease in tensile strength over the same range—indicates that flexural-mode loading is markedly more tolerant of obsidian filler than axial tension, and that the composite remains practically usable in flexure-dominated structural applications (supported panels, lattice elements). The systematic offset of flexural modulus values approximately 6–8% below the corresponding tensile modulus values (e.g., 2.63 vs. 2.81 GPa for neat PLA) is a well-documented print-orientation effect attributed to limited shear transfer between deposited beads and to anisotropic interlayer bonding in FDM-printed PLA [16,17].

Reading all mechanical indicators together strongly suggests that the preferred loading for this system lies at or near 5 wt%. At 5 wt%, modulus and hardness gains are non-trivial, flexural strength is essentially preserved, tensile strength has receded by an acceptable amount, and impact energy remains within a reasonable scatter band. At 10 wt%, the system has crossed from a modulus-dominated improvement regime to a damage-initiation-dominated regime: tensile strength loss has grown, flexural strength has begun to recede, Charpy scatter has expanded markedly, and SEM evidence highlights agglomeration and extended interfacial debonding. This pattern is consistent with the 3–6 wt% optimum window reported by Vidakis et al. [5] for MEX-PLA/glass-filler systems and with the ≈2.5 wt% optimum identified by Aksoy et al. [18] for PLA/perlite. However, since the present design rests on only three composition points (0, 5, 10 wt%), the ≈5 wt% optimum should be regarded as a strong candidate rather than a proven optimum; resolving the true optimum requires a denser composition sweep across the 1–10 wt% interval.

Two clarifications follow. First, the ~6–8% offset between tensile and flexural modulus is already present in neat PLA (2.81 vs. 2.63 GPa) and is a general feature of FFF-printed PLA arising from anisotropic interlayer bonding and limited inter-bead shear transfer; it is therefore not filler-specific. What is filler-specific is the divergence with loading—the tensile modulus continues to rise (3.06 → 3.32 GPa) while the flexural modulus plateaus (3.03 → 3.07 GPa)—which we attribute to the preferential localisation of agglomerates and inter-bead micro-void clusters in the outer-fibre region that dominates the flexural response. Second, the reason this divergence is rarely reported is methodological rather than physical: most mineral-filled MEX-PLA studies report only one modulus (tensile or flexural), so both modes are seldom measured on the same printed material across a composition series. The effect is thus expected to be general for defect-bearing FFF composites, but its magnitude scales with the filler-dependent defect population rather than being intrinsic to neat PLA.

### 4.3. Dual Behaviour in Thermal Stability

The TGA/DTG data (Section 3.7) show an apparently paradoxical bidirectional effect. On one hand, increasing the loading from 5 to 10 wt% produced a ~5–6 °C drop in early-decomposition temperatures T_5_ and T_10_ (322.3 → 316.5 °C and 332.1 → 326.9 °C). On the other hand, the peak decomposition temperature T_max rose by ~4 °C (345.8 → 349.8 °C), the normalized maximum decomposition rate dropped by ~46% (49.6 → 26.9%/min), and the T_20_–T_80_ decomposition interval widened from 9.7 to 23.5 °C.

This dual behaviour reflects competing mechanisms. The downward shift in early-decomposition temperatures can be attributed to an initiation-stage mechanism—moisture or hydroxyl groups physically adsorbed on the obsidian surface catalyzing hydrolysis-like chain-scission of PLA ester bonds, or dispersed particles locally restricting matrix chain mobility and altering relative free volume at higher filler loadings. The slowdown and broadening of the main decomposition event—a 46% rate decrease and a roughly 2.5-fold widening of the interval—are consistent with a barrier/heat-shielding effect exerted by thermally stable silicate particles: dispersed particles diffusively slow the escape of volatile decomposition products from the matrix and modify local thermal conductivity, increasing the effective thermal inertia of the polymer. Similar dual thermal behaviour has been previously reported for PLA/clay nanocomposites [22] and for PLA/ceramic systems [6]. The present study is notable in showing that micron-scale amorphous silicate particles up to 44 µm can also trigger a comparable barrier mechanism on the micro-scale.

The 800 °C residue values (5.41 and 10.35 wt%) match the nominal loadings (5 and 10 wt%) very closely, confirming that obsidian remains thermally stable within the TGA window and provides a quantitative cross-check on the filler content. Referenced to the neat-PLA baseline now measured under the same protocol, T_5_ and T_10_ decrease monotonically across neat → 5 → 10 wt%, confirming that obsidian slightly advances—rather than retards—the onset of decomposition. The calorimetric transitions of neat PLA (glass transition ~58–60 °C, cold-crystallization exotherm ~110 °C, melting ~148.5 °C) agree with nominal PLA behaviour, and the composite melting endotherms (DTA) remain at ~148–149 °C. Obsidian therefore does not measurably modify PLA’s crystallization kinetics or its glass–rubber transition at the loadings examined; its effects are essentially localized in the high-temperature decomposition window. It should be emphasized, however, that this high-temperature barrier action does not translate into improved low-temperature thermal stability: across the compositions studied, the obsidian does not delay—and in fact marginally advances—the onset of early degradation (T_5_ and T_10_), even though it markedly lowers the peak decomposition rate and raises the final residue. The practical ceiling for processing and service should therefore be governed by the slightly reduced degradation onset rather than by the improved peak-rate and char-yield metrics.

This behaviour parallels other silicate/ceramic-filled PLA systems (e.g., PLA/clay and PLA/ceramic composites), in which well-dispersed inorganic particles impede the diffusion of volatile decomposition products and create a tortuous heat- and mass-transfer path; the resulting ~46% reduction in peak decomposition rate and the ~2.5-fold broadening of the decomposition interval are consistent with such a micron-scale barrier mechanism.

Taken together, obsidian is best classified neither as a nucleating agent (the melting endotherm and the low crystallinity are unchanged) nor as a strengthening reinforcement, but as a chemically inert, micron-scale silicate phase that acts as a stiffening filler in the elastic regime, a defect/early-decomposition initiator at the onset, and a diffusion barrier within the main decomposition event. Because FFF-printed PLA is itself sensitive to processing history and post-printing structural rearrangement [10], the neat-PLA reference measured here under the identical protocol is essential: it confirms that the observed shifts are attributable to obsidian rather than to the intrinsic ageing/crystallinity evolution of the matrix.

### 4.4. Micro–Macro Consistency and FDM-Induced Residual Porosity

Methodologically, one of the strongest aspects of this study is that the composition-dependent fracture character produces converging evidence at two independent length scales. At the macroscopic scale (Figure 13), neat PLA exhibits fibrillar extensions, 5 wt% shows cleaner edges, and 10 wt% shows sharp surfaces diagnostic of brittle separation. At the microscopic scale (Figure 14), SEM micrographs verify the same ductile → transitional → brittle progression at the mechanism level: fibrillar drawing and plastic curtain-like folds for neat PLA; residual matrix flow combined with emergent microcracks for 5 wt%; and a fully developed FDM bead architecture with dense microcrack networks, conchoidal obsidian fracture surfaces, and extended interfacial debonding for 10 wt%. The convergence of two independent scales on the same qualitative mechanism establishes a robust scientific basis for the dominant damage mode at 10 wt%—failure of effective load transfer from matrix to rigid filler and the consequent emergence of interfacial debonding as the dominant energy-dissipation pathway during propagation.

The density measurements (Section 3.6) complement these microscopic observations with a quantitative porosity estimate. A void-free rule-of-mixtures prediction yields ≈1.273 g/cm^3^ at 5 wt% and ≈1.320 g/cm^3^ at 10 wt%; the measured values are 1.2291 and 1.2343 g/cm^3^, respectively. This corresponds to a density deficit of approximately 5.8% at 10 wt% loading and points—in good agreement with the periodic V-shaped inter-bead voids clearly visible in Figure 14—to a residual void fraction on the order of 3–6%. This residual void population is interpreted as arising predominantly from process-induced inter-bead and inter-layer voids inherent to FDM, with a secondary but progressively increasing contribution from filler-related defects—interfacial debonding cavities and voids surrounding particle agglomerates—as the obsidian loading rises; a systematic bias of the Archimedes determination is considered unlikely to dominate, since an identical buoyancy protocol was applied to all compositions and the neat PLA reference itself shows only ~1% apparent porosity. The measured density deficit is therefore best regarded as the combined outcome of these mechanisms, dominated by inter-bead porosity. These voids not only account for the density deficit but also constitute a discrete population of pre-existing stress concentrators that act as preferred crack-initiation sites under tensile loading. The porosity–crystallinity–mechanical properties triad in FDM-printed PLA has been explicitly documented by Liao et al. [15], and Wang et al. [16] and Kumar et al. [17] have quantitatively established that the interlayer shear strength is frequently the decisive performance limiter in FDM-printed parts. The wide intra-group standard deviation of the Charpy impact energy in the 10 wt% group (0.411 J, comparable to the mean) and the broad individual value range (0.52–1.00 J, Table 4) provide additional evidence that this residual defect population exerts a stochastic effect on the mechanical response, with sample-to-sample fracture initiation sensitive to local agglomeration, micro-void distribution, and inter-bead boundary quality.

Although a dedicated water-absorption test was not performed, the void population that governs moisture ingress is already quantified: the rule-of-mixtures density deficit (~5.8% at 10 wt%) and the apparent porosity rising from ~1% to ~5% bound the expected moisture sensitivity. A direct moisture-uptake and hydrolytic-ageing study is identified as future work.

### 4.5. Local Resource Advantage, Limitations, and Future Research

To the authors’ knowledge, this study represents the first systematic investigation of obsidian, sourced from the Kars region of Eastern Anatolia, used as a reinforcing filler in PLA matrix composites produced by FDM. Obsidian outcrops in Eastern Anatolia—and particularly around the Kars area—are long known and well documented in geological and archaeometric literature [9,23]. This creates a strategic advantage in raw-material logistics and in the development of regionally sourced value-added filaments. However, geological accessibility does not automatically translate into engineering suitability: reliable scale-up will require source verification, particle size classification, moisture control, and batch-level chemical–mineralogical standardization.

The principal limitations of this study can be summarized as follows. (i) The composition range examined is restricted to two intermediate loadings (5 and 10 wt%), so the evolution of the strength–modulus balance at higher loadings (15–25 wt%) and the possible existence of percolation thresholds have not been resolved. (ii) The obsidian powder was used at a single particle-size fraction (<44 µm), and the effect of bimodal or optimized particle size distributions on strength and ductility has not been explored—even though Dobrosielska et al. [3] have demonstrated that particle size is a decisive parameter in PLA/mineral composite behaviour. (iii) No interfacial modification strategy (silane pre-treatment, MAH-g-PLA compatibilizer) was attempted in this study, although the SEM evidence clearly indicates that filler–matrix coupling is the main performance-ceiling factor. (iv) FDM print orientation was kept fixed, and a systematic study of the influence of print-direction angles, infill, and layer parameters is deferred to future work [16]. (v) The high Charpy scatter in the 10 wt% group constitutes a methodological caveat, and an increased sample count is advisable in follow-up work. Print orientation and raster angle were held constant; their influence on the measured properties is acknowledged as a limitation and a direction for future work.

In light of these limitations, the natural extensions of this study can be prioritized as follows. The primary and highest-payoff intervention is interfacial engineering: surface functionalization of obsidian particles with a silane coupling agent (in particular 3-aminopropyltriethoxysilane, APTES), or addition of a maleic anhydride-grafted PLA (MAH-g-PLA) compatibilizer at low percentages (≈2–5 wt%) [24]. Second, denser composition sampling will be required to resolve the true mechanical optimum. Third, FDM print-parameter optimization—nozzle temperature, inter-bead spacing, layer height, and print orientation—can directly address residual inter-bead porosity and thereby close part of both the density deficit and the Charpy variance gap. Fourth, particle size distribution optimization (bimodal, e.g., 5 µm + 44 µm, or nano + micro hybrids) may suppress agglomeration while homogenizing load transfer. Fifth, geochemical variations across different Anatolian obsidian sources (e.g., Pasinler, Iğdır, Nemrut) should be systematically compared with respect to SiO_2_/Al_2_O_3_ ratio, Fe-oxide content, and hydration state. Finally, micro-CT-based void-fraction analysis, capillary/rotational rheology, and dynamic mechanical analysis (DMA) would elevate the present discussion from a qualitative micro–macro coherence argument to quantitative mechanism evidence.

Several investigations fall outside the scope of this baseline characterization and are identified as priorities for future work: (i) silane (e.g., APTES) functionalization of the obsidian to strengthen the interface and recover tensile strength—the highest-priority direction; (ii) melt rheology/MFI to quantify the processing window; (iii) DMA to resolve the viscoelastic and damping behaviour; (iv) finer composition sampling (e.g., 2.5, 7.5, 15 wt%) to locate an optimum/percolation threshold; (v) moisture-uptake and hydrolytic-ageing testing; (vi) direct interfacial-strength measurement (single-particle pull-out/micro-debond); and (vii) the effect of print orientation and raster angle.

From a practical engineering perspective, the present findings indicate that the 5 wt% Kars obsidian formulation provides modest improvements in stiffness, hardness, and thermal behaviour relative to neat PLA, while preserving tensile strength, ductility, and print compatibility within acceptable bounds. This positions the composite as directly usable in esthetic, flexure-dominant, or low-load structural applications (ergonomic handles, static enclosures, prototyping jigs and fixtures, sustainable design objects). For more demanding mechanical applications, interfacial modification represents the natural continuation of the avenue opened by this study.

## 5. Conclusions

This study systematically characterized the mechanical, thermal, and microstructural behaviour of FDM-printed PLA/Kars obsidian composites (0, 5, 10 wt%). To the authors’ knowledge, this is the first systematic investigation of amorphous volcanic silicate powder, sourced from the Kars region, used as a reinforcing filler in a PLA matrix. The main findings can be summarized as follows.

- Modulus–strength trade-off: The tensile modulus rose from 2.81 to 3.32 GPa (+18.2%) and the flexural modulus from 2.63 to 3.07 GPa (+16.7%) across 0 → 10 wt% loading, while the tensile strength dropped from 57.6 to 47.6 MPa (−17.3%). This classical rigid-filled thermoplastic behaviour is explained by the limited interfacial coupling between the unmodified silicate surface and the PLA matrix.
- Decoupling of tensile and flexural responses: The tensile modulus rose by +8.5% across 5–10 wt% while the flexural modulus changed by only +1.3% and plateaued. Flexural strength fell by only 3.5% at 10 wt% while tensile strength fell by 17.3%. This decoupling stems from the different sensitivities of the two test modes to local particle agglomerates and inter-bead micro-voids; consequently, the composite remains usable in flexure-dominated applications.
- Dual thermal effect: T_5_ and T_10_ dropped by ~5–6 °C across the 5 → 10 wt% transition, while the main decomposition rate decreased by ~46% and T_max rose by ~4 °C. This indicates that obsidian particles act as a dispersed silicate barrier/heat-shielding phase.
- Micro–macro consistency: The macroscopic fracture-edge geometry (ductile → transitional → brittle) and SEM micrographs converge on the same mechanism; the extended interfacial debonding lines observed in Figure 14 at 10 wt% provide direct visual evidence of the tensile strength loss. The density deficit of ~5.8% points to a ~3–6% residual void fraction, in agreement with the inter-bead defects visible in SEM.
- Practical applicability: The 5 wt% obsidian loading provides modest improvements in stiffness, hardness, and thermal behaviour while preserving tensile strength, ductility, and print compatibility within acceptable bounds; it constitutes a directly usable formulation for esthetic, flexure-dominant, or low-load structural applications.

The principal contribution of this work is a baseline characterization of obsidian as a reinforcing filler in PLA. The limitations identified (no surface modification, narrow composition range, single particle-size fraction) define a direct roadmap for future research. Given that the primary future intervention is expected to be silane pre-treatment (APTES) or MAH-g-PLA compatibilization, and given the substantial strength recovery reported for analogous surface-modified volcanic fillers in the literature [18], the structural application window of the Kars obsidian/PLA system may widen, pending experimental verification of the proposed interfacial modification.

## Figures and Tables

**Table 1:** EDS map-sum elemental composition (wt%; *σ* ≤ 0.3). Dashes = below detection.

Element (wt%)	Neat PLA	5 wt%	10 wt%
C	69.6	65.5	67.5
O	30.4	32.1	30.1
Na	–	0.3	0.2
Al	–	0.4	0.5
Si	–	1.4	1.6
K	–	0.3	0.2

**Table 2:** Tensile properties of FDM-printed PLA/Kars obsidian composites (mean ± standard deviation; n = 7). *σ*_max = ultimate tensile strength; *ε* at peak = engineering strain at maximum load; *E* = Young’s modulus from MATLAB R2023b (Version 23.2; The MathWorks Inc., Natick, MA, USA) per-group linear fit on Tracker-based *σ*–*ε* in the 10–30 MPa window.

10 wt%	5 wt%	0 wt%	Property
1.893 ±0.027	2.007 ± 0.012	2.290 ± 0.042	$F_{peak}$ (kN)
47.64 ± 0.68	50.50 ± 0.30	57.63 ± 1.07	$\sigma_{max}$ (MPa)
8.19 ± 0.51	8.67 ± 0.31	10.32 ± 0.33	*ε* at peak (%)
3319.4	3059.6	2807.2	*E* (MPa)
0.9970	0.98938	0.99619	R^2^ of *E* fit

**Table 3:** Three-point flexural properties of FDM-printed PLA/Kars obsidian composites. Per-specimen values are given as mean ± SD. Flexural modulus is reported as the MATLAB best-window linear fit per group on the pooled *F*–*δ* data.

10 wt%	5 wt%	0 wt%	Property
0.401 ± 0.002	0.416 ± 0.002	0.416 ± 0.007	$F_{peak}$ (kN)
12.20 ± 0.31	12.87 ± 0.10	15.27 ± 0.66	$\delta_{peak}\;(mm)$
88.84 ± 0.46	92.18 ± 0.55	92.07 ± 1.53	$\sigma_{f}$ (MPa)
4.07 ± 0.10	4.29 ± 0.03	5.09 ± 0.22	$\varepsilon_{f}$ at peak (%)
3.07	3.03	2.63	*E* (GPa)
0.981	0.972	0.978	R^2^ of E fit

**Table 4:** Notched Charpy impact energies.

Group	n	Mean (J)	SD (J)	Min (J)	Max (J)	*a_k_* (kJ·m^−2^)
**Obs 0**	10	0.640	0.178	0.40	0.90	19.7 ± 5.5
**Obs 5**	10	0.562	0.048	0.50	0.60	17.3 ± 1.5
**Obs 10**	10	0.625	0.411	0.52	1.00	19.2 ± 12.6

Calculation: *a_k_* = *E*/(*b*·*t*), with *b* = 12.7 − 2.54 = 10.16 mm, *t* = 3.2 mm, area = 32.51 mm^2^. Owing to the large standard deviations, the groups are statistically indistinguishable; the ranking (neat PLA > 10 wt% > 5 wt%) is preserved.

**Table 5:** Shore D hardness and density of FDM-printed PLA–obsidian composites.

Composition	Shore D (n = 9)	Density (g/cm^3^, n = 6)
0%	80.22 ± 0.94	1.2283 ± 0.0010
5%	82.33 ± 1.17	1.2291 ± 0.0017
10%	82.44 ± 0.92	1.2343 ± 0.0015

**Table 6:** Thermal properties of PLA samples.

Parameter	Neat PLA	5 wt%	10 wt%
T_5_ (°C)	331.1	322.3	316.5
T_10_ (°C)	341.2	332.1	326.9
T_max_ (°C)	~363	345.8	349.8
Peak decomp. rate (%/min)	— †	49.6	26.9
T_20_–T_80_ interval (°C)	— †	9.7	23.5
Residue @600 °C (wt%)	~1.5	5.41	10.35
T_m_ (°C)	148.5 (DSC)	147.8 (DTA)	149.3 (DTA)
T_g_ (°C)	58–60 (DSC)	— ‡	— ‡
X_c_ (%)	~9 (DSC)	— ‡	— ‡

“~” denotes an approximate value; “—” denotes not detected/not determined; “†” indicates that the value could not be calculated from the DTG curve; “‡” indicates that the value could not be determined from DSC/DTA data.

**Table 7:** Measured tensile modulus of the FDM-printed PLA/Kars obsidian composites compared with micromechanical predictions: the Voigt and Reuss rule-of-mixtures bounds and the Halpin–Tsai model (ξ = 2). Filler volume fractions were computed from = 2.35 g cm^−3^ and *ρ*_PLA = 1.24 g cm^−3^; matrix modulus = 2.81 GPa and filler modulus = 70 GPa. All moduli are in GPa.

Loading	(vol%)	Voigt ROM	Reuss ROM	Halpin–Tsai (ξ = 2)	Measured
5 wt%	2.70	4.62	2.89	3.02	3.06
10 wt%	5.54	6.53	2.97	3.25	3.32

**Table 8:** Modulus–strength response of representative mineral-filled PLA composites.

Filler (Process)	Loading	Tensile Strength	Modulus	Ref.
Glass beads (FFF-PLA)	5–15 wt%	↓	+16%	[1]
Recycled glass fibre (FFF-PLA)	≤25 wt%	−42%	+74%	[19]
Basalt powder (moulded PLA)	≤20 wt%	↓ (~64→55 MPa)	↑ (silane recovers)	[20]
Expanded perlite (moulded PLA)	opt. 2.5 wt%	minor ↓	↑	[18]
Montmorillonite (moulded PLA)	5 phr	+63%	+18%	[21]
Kars obsidian (FDM, this work)	10 wt%	−17.3%	+18.2%	— n.r.

FFF/FDM rows are material-extrusion systems and directly comparable; other rows are conventionally processed literature context. ↑ and ↓ indicate increases and decreases, respectively, relative to the corresponding neat PLA/control material. n.r. = not reported.

## Data Availability

The data presented in this study are available on request from the corresponding author.
